# Exploration of Factors That Affect Engagement With the Experience Sampling Method and Service Users’ Experience of This Within the AVATAR2 Trial: Mixed Methods Study

**DOI:** 10.2196/78204

**Published:** 2025-12-12

**Authors:** Sophie Dennard, Philippa Garety, Clementine Edwards, Andrew Gumley, Oliver Owrid, Lucy Miller, Stephanie Allan, Alison Duerden, Francis Yanga, Chloe Burns, Helena Fletcher, Amy Grant

**Affiliations:** 1 Institute of Psychiatry, Psychology & Neuroscience, King's College London, 16 De Crespigny Park, London, SE5 8AB, United Kingdom, 020 7848 0002; 2 Institute of Psychiatry, Psychology and Neuroscience (IoPPN), King's College London, London, United Kingdom; 3 School of Health and Wellbeing, University of Glasgow, Glasgow, United Kingdom

**Keywords:** digital assessment, psychosis, qualitative, smartphone app, patient and public involvement, interviews

## Abstract

**Background:**

Experience sampling methodology (ESM) is an assessment method used in psychosis research. Symptom severity and gender may be associated with ESM engagement. Exploring qualitative experiences of using ESM among people with psychosis should aid developing more relevant, accessible digital assessments.

**Objective:**

This study aimed to examine factors that could affect engagement with ESM, such as associations of completion rates with age, ethnicity, gender, and clinical severity. It also aimed to explore qualitatively service users’ experiences of using this data collection method.

**Methods:**

Data from 134/207 AVATAR2 trial (ISRCTN55682735) participants were used to evaluate associations between demographic variables, symptom severity, and ESM completion rates. Trial participants were purposively sampled to participate in an interview to discuss their experiences of using ESM or to discuss reasons why they chose not to use it.

**Results:**

Multiple regression analyses of 134 participants found that age, gender, ethnicity, and clinical severity were not associated with ESM completion rates (*F*_5,128_=0.548; *P*=.74). A thematic analysis of 17 participant interviews found 3 overarching themes: *Factors affecting engagement with ESM, Perceived benefits of ESM, and Suggestions for improvement*. These themes described how ESM has multiple benefits for people with psychosis, including increasing knowledge and awareness of mental health. ESM was straightforward and easy to use; however, engaging in other activities, experiencing positive symptoms, little experience using technology, and trial involvement impacted engagement. Participant’s decision to use ESM could be influenced by concerns about security and privacy.

**Conclusions:**

Recommendations are made on how engagement with ESM can be improved, making it easier to use this method with this population, including providing increased support or training when using digital-based assessment or intervention as well as providing information on how digital data are used and recorded.

## Introduction

The experience sampling methodology (ESM) is an assessment method that prompts individuals to complete repeated, in-the-moment, questionnaires several times a day [1]. This methodology allows researchers and clinicians to obtain information about an individual’s everyday life experiences, with less reliance on retrospective memories. ESM has therefore been used in a wide variety of research and clinical settings in populations of individuals with diagnoses of schizophrenia and psychosis [2-4].

Research has indicated that the validity of ESM may be impacted by different factors, including the items used [5]; however, it may also be partly affected by levels of engagement of those completing the assessments. Although the findings are inconsistent, some factors affecting engagement with ESM have been investigated in people with psychosis. Research has found higher rates of engagement among females, older participants, and people identifying as White [5-7]. Furthermore, studies have reported that those with increased symptom severity are more likely to drop out of using assessment tools such as ESM or have lower levels of engagement than those without a mental health diagnosis or individuals with less severe symptoms [8,9]. Although some findings suggest that ESM engagement may be impacted by particular demographic or clinical severity factors, these findings have not been robustly replicated and warrant further investigation.

There is a growing interest in evaluating the impact of psychological interventions on patients’ everyday life. One way in which this has been done is through the addition of an ESM component to the randomized controlled trial (RCT) design of research trials. Research focused on the area of psychosis or schizophrenia spectrum disorders has been leading in this field of incorporating ESM into research designs [10-13]. However, studies report low completion rates and questions have been raised about how to best integrate this data collection method in a way that maximizes valid and reliable outcome assessment in daily life, while minimizing burden. This study is the first that addresses this dilemma directly through in-depth evaluation of this experience, conducting qualitative interviews with trial participants who have been invited to take part in an embedded ESM study.

Compared with research investigating factors that impact engagement, there is still relatively little research examining individuals’ experiences of using ESM qualitatively. There are also relatively few studies that have aimed to identify reasons why individuals with psychosis may decide not to use mobile apps, of which ESM is often delivered, within the context of research or therapeutic interventions. Existing research suggests that concerns around the privacy of data collected could impact individuals’ willingness to engage with assessment methods such as ESM, as well as increased feelings of paranoia and rumination when being asked to complete repeated questionnaires [14,15]. Gathering the experiences of people using ESM may therefore be particularly useful in the development or improvement of technology to be used in research and treatment of psychosis. This study firstly aimed to examine factors that affect engagement with ESM, by examining quantitative associations of completion rates with age, ethnicity, gender, and clinical severity, and secondly explored qualitatively service users’ experiences of using this data collection method.

## Methods

### Procedure

The original purpose of the ESM study was to assess voice-hearing experiences in everyday life as secondary outcomes in the AVATAR2 trial [16]. Demographics and the Psychotic Symptom Rating Scales (PSYRATS) scores were obtained through assessments with research assistants in the baseline assessments during the AVATAR2 study [17]. These demographics and scores were then recorded on a database which could be accessed by those completing the current ESM study. The baseline assessment data used within this study included data from 207 participants recruited between January 2021 and April 2022.

Purposive sampling was used to select participants for qualitative interviews. Participants were selected in 3 groups based on terciles of ESM completion and a fourth, ESM opt-out, group. This was done in order to generate a range of experiences of using ESM. The groups were participants with low (18% and below), medium (19%‐54%), and high (55% and above) completion rates, as well as participants who chose not to use ESM.

### Ethical Considerations

This study was linked to the AVATAR2 trial (ISRCTN55682735), which is a multicenter UK-based RCT. The AVATAR2 trial is investigating whether the development of dialogue with a visual representation of a voice can help reduce distress, frequency, and severity of voices in those who experience auditory hallucinations [3]. Additional research ethics approval for this study was granted in January 2022 by the London-Camberwell St Giles Research Ethics Committee (reference 20/LO/0657) through submission of a substantial amendment to the original ethical approval. A protocol for this study, along with all study documents, has been published on the Open Science Framework [18].

AVATAR2 participants who had consented to be approached for further research were contacted via telephone and asked whether they would be interested in participating in a qualitative interview. All of the participants had already completed their participation in the AVATAR2 trial prior to this study. Interviews were held either online via Microsoft Teams or in person, and all participants gave informed consent and were able to opt out at any point within the study. Participants were paid £20 (US $26.16) for their participation in the interview. All interviews were transcribed, anonymized, and checked for accuracy prior to analysis. All participants provided with informed consent, including that anonymized quotes may be used within a publication of the study findings.

### Measures

#### Demographics

Demographic variables were information regarding participants’ age, gender, and ethnicity.

#### Clinical Severity

Clinical severity was determined by overall baseline assessment scores on the auditory hallucinations’ subscale of the Psychotic Symptom Rating Scales—Auditory Hallucinations Subscale (PSYRATS-AH) [19]. The PSYRATS-AH is made up of 11 items and scores can range from 0 to 40, with higher scores indicating higher clinical severity.

#### ESM Completion

Participants who opted in for the ESM assessment were sent questionnaires via the smartphone app M-Path 10 times a day for 6 days [20]. The questionnaire included items assessing affect, voice-hearing experiences, social interactions, and activity. The completion rate was defined as the percentage of questionnaires that were 100% complete, and this decision was made as saving incomplete questionnaires through the M-Path app had implications for the reliability of the data export.

The ESM data were collected at 3 timepoints—baseline (prior to randomization), 16 weeks, and 28 weeks—and at these latter 2 timepoints, the ESM period took place after the trial assessments. The same participants were asked to complete these assessments at each time point. These assessments were collected at 3 time points in line while completing other trial-related data. Following the ESM assessment period, participants were asked to complete a debrief questionnaire. The debrief questionnaire was administered via phone call by a research assistant to collect structured feedback on the experience of the ESM week and took approximately 5 minutes to complete. This questionnaire was developed during a previous ESM study conducted with people with a diagnosis of schizophrenia and is not coproduced [1]. The questionnaire asks about people’s experience of disruption during the week due to the ESM questionnaires, how easy they found it to complete, adequacy of support provided, and whether they enjoyed the experience, and there are also open text boxes for more detailed feedback. A copy of this questionnaire can be found in Multimedia Appendix 1.

### Patient and Public Involvement and Development of Interviews

Qualitative interviews were administered using topic guides that were developed for the study. The interview topic guides were developed in collaboration with the trial’s patient and public involvement (PPI) consultants (people with lived experience of psychosis who were involved in a range of activities supporting the trial). Preliminary drafts of the topic guides were developed by the first author. These guides were then reviewed by research assistants and PPI consultants. Feedback on these topic guides was given verbally through a virtual meeting with the first author (SD), as well as written feedback provided by email. Such feedback enabled changes to the wording of the topic guides with resulting improvement in accessibility. Final topic guides included open-ended questions designed in order to explore the following areas: participants' involvement in AVATAR2, familiarity with mobile phones, and reflections on their use of ESM. A walk-through of the M-Path app was provided to participants who opted in to using ESM in order to support with their reflections on using the app [21]. For those who opted out of ESM, a walk-through of the AVATAR2 consent form where they would have been asked about participating in the ESM part of the study was conducted.

Interviews lasted between 21 and 64 minutes. Interviews were conducted by the lead author (SD). Of the 17 interviews, 11 (64.7%) were coconducted by a PPI consultant. Three PPI consultants received 3 hours of training in conducting qualitative interviews. PPI consultants were paid in line with NIHR INVOLVE guidelines for their time [22].

### Reflexivity Statement

The lead author is a White British, female clinical psychologist with prior experience of working clinically with people with psychosis. She has particular interests in understanding the experiences of people with psychosis in order to improve the quality and availability of treatments for this population. As such, the lead author’s epistemological position is most closely aligned to a “critical realist” perspective [23]. This meant that a flexible deductive approach was used throughout the research process. For example, preexisting research knowledge was held in mind when defining the research question and in developing the topic guide for the semistructured interviews. This flexible approach continued throughout the data analysis process, where both information from the data and feedback from the PPI consultants informed preliminary and secondary codes, as well as themes and subsequent interpretation.

The lead author had not previously had any contact with the participants of the AVATAR2 trial. The other authors were all members of the AVATAR2 research team ranging from PPI consultants to principal investigators with varying degrees of contact with participants of the trial. All of the PPI consultants who were involved within this study were individuals with lived experience of psychosis, some of whom had previously been participants in the first AVATAR research trial [24].

### Analysis Methods

An a priori power analysis of ESM engagement indicating a total sample of 108 or above would be necessary to detect associations at a medium effect size with 90% power (*f*^2^=0.15). IBM SPSS (version 28.0; IBM Corp) package was used to analyze the associations between engagement and other variables and for descriptive statistics. A multiple regression was conducted to determine whether there were any associations between demographic variables and clinical severity with ESM completion rates.

For the qualitative investigation, all interviews were transcribed and analyzed by the first author (SD) using a reflexive thematic analysis approach [25]. All of the transcribed data were thoroughly read in order to increase familiarity with it. All data were reread in order to identify initial codes. A further read of the data was conducted to identify further, more interpretative codes. These codes were used to help generate preliminary themes. A hybrid approach was taken, deductive in that prespecified research questions guided the analysis, with an inductive, data-driven approach to how the themes that answered these questions were generated. The focus was on semantic codes, a description of explicit meanings provided by participants regarding their experience.

Preliminary codes and themes of data from half of the interview transcripts were reviewed by the PPI consultants who had conducted interviews. PPI consultants provided feedback and interpretation in relation to the initial codes and themes. This feedback related to how the themes related to their own experiences and how the wording of the themes was able to capture the different preliminary codes. This feedback informed not only how the final themes were worded but also which information within the themes would be most important to highlight. For example, PPI consultants spoke about their own mental health experiences and how these could impact their usage of ESM during our interviews, and this was foregrounded through the selection of quotes. These preliminary themes evolved with further analysis of the remaining interviews. Themes generated from all of the interview data were again reviewed and checked by PPI consultants who coconducted the interviews.

Data sufficiency was ensured by reflecting on the content as interviews were conducted and through discussion with PPI cointerviewers and the research team agreeing that sufficient information power had been generated to address the research questions [26]. Rigor was ensured through strategies including triangulation between lead researcher, wider research team, and lived experience experts when developing themes in the analysis, and reflexivity being considered at all stages with an awareness of potential biases that may influence the findings.

## Results

### Factors Affecting ESM Completion

The responses to the debrief questionnaire can be found in Multimedia Appendix 2. The findings from this questionnaire show that the majority of participants reported minimal disruption or deviation from usual routines during the ESM week and were able to access the assessments on the app with the equipment, training, and support provided.

Demographic information and baseline assessment data including the PSYRATS were obtained for 207 participants. Of these participants, 134/207 (64.7%) consented to take part in the optional ESM component of the assessment. The mean ESM completion rate across the 134 participants who consented to ESM assessment was 39.1% (SD 28.5). The demographics of the whole sample (N=207) as well as the ESM (N=134) and interview sample (n=17) can be found in Table 1. Information about which RCT treatment group the interview sample was assigned to within the AVATAR2 trial is also included in Table 1. The psychiatric diagnoses of the whole sample can be found in Table 2.

**Table 1.** Demographic variables for participants within the whole, experience sampling methodology, and interview sample.

Variable	Whole sample (N=207)	ESM^a^ sample (n=134)	Interview sample (n=17)
Age (years), median (range)	38.10 (18-70)	36.34 (18-66)	44.47 (22-62)
Sex, n (%)			
Male	123 (59.4)	75 (56)	10 (58.8)
Female	80 (38.6)	56 (41.8)	7 (41.2)
Other	4 (1.9)	3 (2.2)	0 (0)
Ethnicity, n (%)			
White	125 (60.4)	91 (67.9)	9 (52.9)
Black Caribbean	16 (7.7)	8 (6)	2 (11.8)
Black African	12 (5.8)	8 (6)	3 (17.7)
Black Other	4 (1.9)	3 (2.2)	0 (0)
Indian	8 (3.9)	4 (3)	0 (0)
Pakistani	8 (3.9)	5 (3.7)	0 (0)
Chinese	1 (0.5)	0 (0)	0 (0)
Other	33 (15.9)	15 (11.2)	3 (17.7)
PSYRATS^b^ score, median (range)	29.94 (13-41)	29.38 (13-40)	27.82 (13-35)
RCT^c^ treatment group, n (%)			
Treatment as usual	N/A^d^	N/A	7 (41.2)
Brief	N/A	N/A	5 (29.4)
Extended	N/A	N/A	5 (29.4)

a ESM: experience sampling methodology.

b PSYRATS: Psychotic Symptom Rating Scales.

c RCT: randomized controlled trial.

d N/A: not applicable.

**Table 2.** Psychiatric diagnoses of participants within the whole sample.

Diagnosis	Total count	Total %
F20 Paranoid schizophrenia	83	39.7
F22 Persistent delusional disorder	2	1.0
F23 Acute and transient psychotic disorder	3	1.4
F24 Induced delusional disorder	1	0.5
F25 Schizoaffective disorder	18	8.6
F28 Other nonorganic psychotic disorder	1	0.5
F29 Unspecified nonorganic psychosis	75	35.9
F31 Bipolar affective disorder	5	2.4
F32.3 Severe depressive episode with psychotic symptoms	19	9.1
N/A^a^	2	1.0

a N/A: not available.

A multiple regression was carried out to investigate whether age, gender, ethnicity, and clinical severity could significantly predict ESM completion rates within the ESM sample. The results of the multiple regression indicated that the model was not a significant predictor of rates of ESM completion (*F*_5,128_=0.548; *P*=.74). Coefficients and significance values for each hypothesized predictor can be found in Table 3.

**Table 3.** Results of multiple regression for predictors of experience sampling methodology completion.

Predictor	*B*	SE	β	*t* test (*df*)	*P* value
Age (years)	0.312	0.205	.137	1.53 (10,123)	.13
Sex					
Male versus female	0.736	5.27	.013	0.140 (10,123)	.89
Male versus other sex	0.466	17.026	.002	0.027 (10,123)	.98
Ethnicity					
White versus other ethnicity	−3.124	5.395	−.051	−0.579 (10,123)	.56
Clinical severity	−0.109	0.501	−.020	−0.217 (10,123)	.83

### Qualitative Exploration of Experiences of Using ESM

In total, 59 participants were contacted to ask whether they would be interested in participating in the study. Overall, 17 participants agreed to take part in the study and completed qualitative interviews. Table 4 shows the number of participants in each of the different ESM completion rate groups. The thematic analysis resulted in 3 overarching themes and 9 subthemes. The overarching themes were *Factors affecting engagement with ESM, Perceived benefits of ESM, and Suggestions for improvement*. Themes were generated using interview responses from across the 4 ESM completion groups (low, medium, high, and no ESM). Specific quotes accompanied by information on which group the participant was drawn are included. Further participant quotes related to each overarching theme and subtheme can be found in Multimedia Appendix 3.

**Table 4.** Range of experience sampling methodology completion across groups and number of participants per group.

ESM^a^ group	Participants across whole sample (N=207), n (%)	Participants interviewed (n=17), n (%)	Completion rate range, %
Low	46 (22.2)	4 (23.53)	0‐18
Medium	42 (20.3)	4 (23.53)	19‐54
High	46 (22.2)	5 (29.41)	55+
No ESM	73 (35.3)	4 (23.53)	N/A^b^

a ESM: experience sampling methodology.

b N/A: not applicable.

### Factors Affecting Engagement

This first theme relates to previous experiences, individual factors, or factors related to ESM which made it easier or harder to engage with. This theme comprises four subthemes: (1) integrating ESM into daily life, (2) impact of symptoms, (3) experience with technology, and (4) experience of therapy and research.

1. *Integrating ESM into daily life*: For a number of participants, the questions felt quick and easy to answer and became part of their routine. The format of the questions also helped make the questionnaires straightforward to answer, which could be particularly helpful for those who have dyslexia.

*Well, it was fairly straightforward to answer the questions*.

[P001, line 35, High]

*Yeah, just I think it just became a routine*.

[P017, lines 92-93, Low]

*I have dyslexia. And so when I am reading like a book or something, it’s quite overwhelming with everything that’s around it. But because it focuses on the question and then it’s just it just comes up where it shows you and like, it’s just like a scale. It’s a lot easier to use a scale than umm explain at times I am...And that’s quite helpful*.

[P009, lines 332‐335, Medium]

Participants reported that the number of times in which they were prompted to answer the questionnaires each day within the sampling period could feel repetitive and tedious. For some, the knowledge that it was only for a limited period of time increased their motivation to complete the app; however, that engaging with this longer term would not be sustainable. This may have been particularly the case for those who had higher completion rates and therefore felt that doing the questionnaires as frequently per day could not happen over a longer period of time. For a participant who did not use ESM but was informed of the number of times the questionnaires are sent through in a day, they reported that the frequency of this would have been too much.

*It was a bit a little bit repetitive*.

[P017, line 77, Low]

*It’s quite time consuming. You know it makes quite a lot of demands in terms of how often and frequently you have to use the app*.

[P001, lines 16‐17, High]

*I don't think I would enjoy 10 times a day, six days*.

[P011, line 150, No ESM]

A number of participants reported that their ability to complete the app was impacted if they were engaging in other routine tasks or activities. Several participants commented on how they managed this by completing the app at a time that was convenient to them. Although this may have been good for them and made it easier to integrate into their day-to-day lives, this could have impacted their overall completion rate.

*I still had meetings with CPN’s at the time. I had other meetings that I had to. That I had to attend...I mean you can’t do it as and when the notifications spring up because you have a life to live*.

[P001, lines 106‐107, High]

*Well, the only trouble was it would ping when I’m driving the car it would ping. You know when you sometimes you just can’t respond at that at that time, whereas at work or so, yeah, that was the only problem that I couldn’t. I couldn’t answer when you know exactly when it pinged*.

[P008, lines 86‐88, Medium]

*Umm, I think I just checked it when I got home...I didn’t do anything outside*.

[P014, line 133, Low]

The notifications and questionnaires could for some feel as though they were impacting their ability to relax. This was particularly the case for notifications that occurred in the afternoon or evenings when they were more likely to be at home. Receiving notifications from the M-Path app was reported as a potential factor in ESM completion levels, as some participants reported not having received any or not hearing them due to their sound being turned off. Those who were in the high completion groups may have been thinking more consciously about the notifications due and therefore checking their phones more regularly, or perhaps were more familiar with the notification settings on their phone than those with low completion rates.

*I feel like it’s like A. Bit of a weight to carry when I’m out and about it’s it’s it’s not. It’s like a? whats that word when something is. Not a liability...A burden that’s it*.

[P003, lines 266‐268, High]

*I didn't get the beeps*.

[P013, line 131, Low]

I think maybe once or twice I missed it, you know, but only because I’m missing notification and then I’d label come my phone and maybe like oh **** went off an hour ago, you know? And then go ahead and fill it into that point.

[P007, lines 132‐133, High]

2. *Impact of symptoms*: Participants reported that feeling low in mood could impact their motivation and ability to complete the app. The sleeping patterns of some participants were reported to impact the answering of the questionnaires. For some, this was due to the side effects of the medication that they were prescribed which could impact how they felt, especially in the mornings. For others, the voices they hear were mostly experienced at night, which affected their sleep, and therefore it may be that time was spent in the day catching up on this sleep and therefore impacting on responding to the M-Path notifications:

*I wasn’t always in the mood I felt. Miserable. Uh. Uh, whereas other times, I didn’t mind doing it...I think it’s basically it’s essentially mood based*.

[P016, lines 128‐129, Low]

*Yeah, you know, I mean, I’m up at like, two, three, four in the morning. You know what I mean? Pacing up and down*.

[P005, line 125, Medium]

*I wasn’t able to the morning ones because in that time I was on medication, so I was sleeping quite a lot*.

[P009, lines 126‐127, Medium]

A number of participants reported their voice-hearing experiences affected their ability to complete the app due to having difficulties concentrating or due to the voices encouraging the participant to not respond at that time. Experiences of paranoia and beliefs that their thoughts and the questionnaires were connected could also impact some participants’ ability to engage with ESM. For some, the voices may not have been present when answering the questionnaires, which for one participant was felt to be due to the voices having an awareness of the app. For others, not hearing voices when responding to the questionnaires was more challenging due to difficulties connecting with their experiences. This appeared to be particularly for people in the high responder groups, whereas for those in the lower responder or no ESM group, the presence of voices while using the app was thought of more.

*I would there was a few times where umm I would be having mm I would be talking to <voice> and we would be focusing on my <job> and the beep would go off, but <voice> would just tell me to just like to focus on our our work*.

[P009, lines 90‐92, Medium]

*Yeah, like they were connected. A couple of times my my brain went that way...I’m getting paranoid about <therapist> now, thinking <therapist> set me up, you know, all that stuff was like for hours, I was debating about it*.

[P005, lines 210‐214, Low]

*But then sometimes I’d like rush through it because I’m like, I don’t know if I can, if I’m really like, if any of this really connects with what’s happening right now*.

[P007, line 99‐100, High]

*Obviously it depends on how you're feeling now that particular time. Whether the voices were intense or not*.

[P011, line 180, No ESM]

A number of participants reported how difficulties with their memory could impact on being able to remember aspects of the trial or their experience of using the app. Others reported that their memory also impacted their ability to remember to engage with the app and answer the questions during the data collection period.

*But at times I don’t remember. Where I would complete the the beep and then I would look at it and the other sections beforehand was done, but I don’t I don’t remember doing that*.

[P009, lines 354‐356, Medium]

*I think it was just about remembering to answer the questions within a certain time period*.

[P017, lines 259-260, Low]

3. *Experience with technology*: Although all of the participants interviewed reported currently owning a smartphone, the degree to which they were familiar or found using technology easy differed across the different completion groups. Specifically, the majority of participants who specifically reported to be comfortable and knowledgeable using smartphones were in the high or medium groups. Conversely, most participants in the No ESM group reported either not being very knowledgeable or confident in using technology or reported a dislike of using it. This could in part be impacted on their views of technology and specifically the link with technology and their symptoms.

*To tell you frankly, yeah. I don't like to use technology*.

[P010, line 165, No ESM]

*I’ve used them for a while. I know how to use the apps and they’re fairly straightforward. I’m I’m not really a technophobe so*.

[P001, lines 11‐12, High]

*Yeah, but as I said, I don’t like doing all that on the phone, and I couldn’t. I’m illiterate on the Internet*.

[P005, lines 75‐76, Medium]

Several participants reported requiring support in using smartphones. This, for some, was in the context of day-to-day life in using different phone features, whereas other participants spoke of needing additional support in setting up and using the app. Several participants reported experiencing concerns regarding technology including uncertainty around who may be accessing their data; however, for a number of participants, these concerns did not impact their ability to continue using the app. However, for those who chose not to use ESM, concerns about technology appeared to be a significant factor in their decision to opt out of using ESM.

*If I am shown it is okay. Yes I can't do it myself but if I am shown*.

[P014, line 25, Low]

*I worry about being like intercepted and. So I mean, things like that, so I have to. I would have kind of like. Umm and ahhing about it and in the end I just got on with it*.

[P003, lines 32‐34, High]

*I wouldn’t want the phone health questions falling into someone else’s hands*.

[P011, line 95, No ESM]

*I don't really trust technology though, because I wonder if who’s looking at it, you know*.

[P012, line 107, No ESM]

4. *Experience of therapy and/or research*: Some of the participants spoke about their feelings of happiness or gratitude for being part of something new and something that could benefit others in the future. Participants also demonstrated a willingness to engage in something whereby they could learn new ways of managing their mental health.

*I’m happy to to to use and if if it’s, if it’s part sort of future treatment then yeah I’m all for it*.

[P017, lines 108‐109, Low]

*I felt really lucky to have that there’s quite new. It’s quite different and it’s just rolling it out. I felt quite happy to do it, yeah, I felt happy about it*.

[P003, lines 5‐6, Medium]

Having signed up for the AVATAR2 trial and opting in to use ESM for some motivated their engagement and completion of the app. For some participants, this meant that completing the app was a way that they could help the trial or researcher that they had been engaging with. For others, the knowledge that they have agreed to participate and were being paid to participate was an influencing factor in ensuring that they continued to engage and complete the app.

*It was like a game but plus <researcher> was paying me, I was like I have to do, <researcher> was paying me money to do it*.

[P003, lines 92‐93, High]

*I felt it was something I had to do to help...So that is why I answered the questions*.

[P015, line 166, High]

*Right at the very beginning, there was a lot of information, a lot. (Yeah) You know lots of questions, lots of there’s lots of that, you know? And. And yeah, I mean, beginning always really apprehensive as to what’s gonna happen, aren’t you? So you know, it’s not. I mean, I think I’ve got probably a bit overwhelmed with the amount of information that was probably given to me on that day*.

[P006, lines 280‐285, No ESM]

### Perceived Benefits of ESM

This second theme relates to the potential benefits that can result from using ESM. This theme comprises two subthemes: (1) access to increased support and (2) enhanced awareness.

1. *Access to increased support*: Participants reported that ESM provided a space whereby they could express their feelings and feel as though they were talking with someone about their experiences. This space was felt to be particularly helpful for those who felt worried about feeling embarrassed, not being understood by others, or where they did not want to burden others by talking about their experiences. Considering ESM is often not used as a therapeutic intervention, yet participants felt it was a space in which they could share their experiences, could be reflective of the context in which ESM was used. Being part of an RCT may enable participants to feel as if their responses were going to be seen by the research team.

*I was able to express myself. (Yeah) Where I wasn’t holding all of that and were I when I felt low, I was able to tell someone*.

[P009, lines 265‐266, Medium]

*Umm and the app itself, because it was an app and there was no direct interface with another person there, there wasn’t really any chance of it being awkward...You know, I’m I’m just an anonymised person at the end of the lane. So there there’s not much scope for embarrassment about it. (Umm) So like like I found that quite easy to do and I like it*.

[P001, lines 156‐160, High]

Other benefits of ESM included the helpfulness of concentrating on answering questions as a distraction from hearing voices or dissociative experiences. As well as providing a distraction from voices, the use of ESM could itself be considered as a coping tool that could reduce the amount of distress they were experiencing and to help them feel grounded.

*I don’t think I ever had. I had that because maybe because I was concentrating. You know, with me, the voices, if I’m concentrating on doing something. Yeah. (ok) Yeah. Then then I don’t really. I don’t really. So I don’t think to be fair, I don’t think I am. Yeah, I don’t think I heard them when I was answering*.

[P008, lines 370‐372, Medium]

*And it it did help ground cause. Answering questions about. Like. If the voices stressing me or. Like really thinking about it helped*.

[P009, lines 143‐144, Medium]

2. *Enhanced awareness*: Participants reported that the app provided them with an opportunity to increase awareness of their mental health and to provide them with space where they could spend time reflecting on their experiences, which could be helpful in being more present and checking in with reality. Participants also spoke of being able to increase their awareness and understanding of links between their mood and voice-hearing experiences.

*I got me thinking about my experience regularly and. Yeah, maybe being more self-aware about what was happening to me*.

[P007, lines 278-279, High]

*Umm did did the voices talk to me more often today did? Did what I do yesterday work today? It kind of raises those kind of questions. It is is introspection reflectiveness*.

[P016, lines 87‐88, Low]

Some of the participants reported that their increased awareness enabled them to think of activities they could engage in to improve their mood, as well as to reflect on different triggers that could have impacted their mood and voice-hearing experiences. Answering the questions related to voice-hearing experiences enabled some participants to reflect on the relationship with their voices and how this may have changed over time. For some, this provided them with a sense of feeling more in control of their experiences. This was an experience that participants across the completion groups experienced and therefore could indicate the benefits of the app regardless of times they interacted with it.

*I suppose this app makes me kind of. (Umm) Become more aware of what I need to do so I’m really not feeling relaxed and then I realise it from this question. Then I could do something about it because sometimes I don’t know how I feel, until it’s it’s there in black and white*.

[P003, lines 161‐163, High]

*Sometimes it is just the right focus and acknowledging that it is just voices*.

[P014, line 149, Low]

*It just I keep saying this, don’t I? It just puts you in control. Umm, because you can expect to feel a certain way. At a certain time, so it it just allows you to say, OK, I’m gonna feel rubbish this day, but it’s because XYZ and yeah, it just it just puts you in control, which makes life so much easier rather than feeling, you know, a victim and and and down underdog and you know, all that kind of thing*.

[P008, lines 163‐166, Medium]

### Suggestions for Improvement

This third and final theme relates to feedback given by participants on how ESM could be improved. This theme comprises three subthemes: (1) specificity and customization, (2) need for further support, and (3) access to solutions and strategies.

1. *Specificity and customization*: A number of participants reported a desire for questions to be more specific and more tailored to personal experiences. For some, the questions seemed to lack clarity and therefore made them harder to answer the questions. A number of participants reported that they would have benefitted from a space where they could write free text responses to be able to more accurately describe their experiences. For others, the opportunity to write free text was thought to be helpful in being able to keep a record or track of what they were doing before experiencing psychosis symptoms.

*I mean, sometimes like, you know how the experience well for me and for other people, I guess the experience is very individual of hearing voices and having these sorts of experiences. So sometimes like the generic questions were like, really hard to see how...sometimes they just didn’t really relate to your experience*.

[P007, lines 76‐78, High]

*The questions were simple...so I did it the best I could. But there wasn’t anywhere that I could write a little message or a note*.

[P014, lines 101‐102, Low]

*If there could be like a wee box or where they could write what they’re doing, so they’re able to like keep track of what they were doing before*.

[P009, lines 336‐337, Medium]

Some of the participants reported wanting to be able to customize the number of notifications they receive or to target them at certain times in the day when it would be more convenient and fit them in with their routine. For a participant who chose not to use ESM, and whose biggest concern was the security of the app, having the opportunity to increase the security of the app, for example, being able to input a personalized pin to access the app, was felt to increase the likelihood of their future use of similar apps. They also suggested an option to be able to delete the app from another device if their phone was to be stolen, which could be more of a concern for those who chose not to use the app and may be linked to concerns around privacy.

*If people could choose if they wanna know once...once a day, twice a day, six times a day. It would be good to choose*.

[P003, lines 175‐176, High]

*Maybe an app where you can delete it from a distance*.

[P011, line 116, No ESM]

2. *Need for further support*: Participants across different ESM completion groups reported that being offered more support in answering questions and receiving reassurance that what they were submitting was correct would have been beneficial. Those within the lower completion groups could have felt uncertain and therefore did not answer the questions, whereas those in the higher groups may have answered regardless but would have benefited from being debriefed. Although the desire for further support with completing the app was reported amongst different participants, a reluctance to seek support was also highlighted due to concerns that this would reveal their identity and treatment condition within the trial.

*Probably it might have been a good thing if I’ve been able to...I had a debriefing meeting at the end of it*.

[P001, lines 83‐84, High]

*Umm. Well, because I kept on hearing the young man as well. Umm, it put me into a difficult position because I had to, like, overcome the main voice. (Right). And then I have this other voice. Umm, you know, like being really, really disruptive in my life, you know? (Yeah). I felt like I needed encouragement*.

[P013, lines 229‐231, Low]

*Yeah. Because I wasn't sure. (OK). And I didn’t want to let anyone know that I was getting the therapy right*.

[P013, line 236, Low]

Some participants reported that it would be helpful to have an app whereby their care team can access the information in order to have a better understanding of their mood. The integration with care teams was felt to be particularly helpful in relation to the safety of the individual they are supporting in order to monitor any reduction in mood which could indicate increased risk to self. The inclusion of family and friends was also suggested by participants as an opportunity for discussion and increasing understanding of their experiences.

*I do think it would be quite helpful to have umm the app where you. For example, like your CPN or mental health team or your doctor being able to check how (Umm) how your mood has been*.

[P009, lines 320‐322, Medium]

*It could be interesting, even maybe to include someone who’s, if if I’m not home, someone comes to visit me my <relative> you know, maybe even to ask <relative> or, you know, would you go through the questions with me? (Yeah). Involve someone*.

[P017, lines 277‐279, Low]

3. *Access to solutions and strategies*: Some participants reported that it would be useful to have the option to see their past answers or be provided with a summary of daily responses. The ability to access summaries *or* past entries was reported to be useful in aiding their memory of their answers on that particular day.

A little note at the end or at the end of each question to say umm, just to what you are doing.

[P014, line 207, Low]

One participant highlighted that the app was able to only collect answers to questions and did not give the option for providing solutions. Due to a lack of solutions provided within the app, participants made a number of suggestions on how the app could be improved to provide an increased opportunity to self-manage and have easy access to different coping strategies. These suggestions tended to be made by those in the high completion group, which could be indicative of more interaction with the app and therefore increased insight into ways in which it can be improved.

*The thing that this is just looking for answers. It’s not giving solutions. It’s just looking for answers*.

[P003, lines 112-113, High]

*A basic care plan that people make when they’re well and they kind of put down how they would like to be treated when they’re unwell for example, what’s worked in the past like double up on antipsychotics just for two weeks or go and have a massage or watch a Disney film or have a hot bubble bath. Visit someone go to go to mosque or church. Whatever you know like so that the person is been involved in...in the solution in the in the recoveries and stuff that they know that works*.

[P003, lines 128‐131, High]

## Discussion

### Principal Findings

This study aimed to identify factors that could affect engagement with ESM and to explore participants’ experiences of using ESM within the context of the AVATAR2 trial. ESM completion rates were not found to be associated with demographic and clinical severity factors. These results are encouraging and suggest that ESM can be engaging across a diverse user group. Participants were able to report a number of factors that could either facilitate or be a barrier to using ESM. Participants also reported a number of suggestions in order to enhance their experiences of using the app. Due to the nature of ESM when used as an assessment tool which requires the data gathered to be reliable and valid, certain suggestions mentioned by participants, including the customization of notifications, may be problematic and result in bias. Although these suggestions may not be applicable for ESM when used as an assessment tool, the findings could be useful in the development and implementation of other forms of digital tools. For example, the use of ecological momentary intervention has been investigated and used within people with psychosis to support with symptom monitoring and self-management within clinical settings [2,12,27].

All participants who were interviewed in the current study reported owning a smartphone, which is consistent with previous findings related to increased smartphone usage within psychosis populations [28,29]. Despite this consistent ownership, the degree of knowledge and confidence in using these devices differed across the sample. Therefore, owning a smartphone does not necessarily equate to having the knowledge and skills in using them, which has been identified in previous research [30,31]. It is therefore important that individuals are also offered opportunities for training to increase their knowledge and skills of using technology. This can help to make it easier for them to use and to reduce barriers in accessing digital mental health support. Previous research has highlighted the importance of effective briefing and communication while engaging with ESM and the positive impact on completion rates when receiving such support [32]. This increased support may be particularly useful for people currently experiencing positive symptoms, which were reported by some participants in this study as a factor affecting their ability to respond to the questionnaires.

Concerns about privacy and security were raised within the current study, similar to findings in previous research [33]. These concerns were notably expressed by the majority of participants who chose not to use ESM. Having more information about the app and additional security may be helpful for some, but for others, it may not be sufficient to increase their willingness to use ESM due to the extent of their concerns about technology. Therefore, in some instances, the use of digital data collection methods such as ESM is not going to be acceptable for all, and it is important that other methods are also used for those who do not wish to use technology. This also applies to other digital tools and interventions whereby people with psychosis may still like to access support but would like to do so without having to rely on technology. It is worth highlighting that concerns about, or trust in, technology are not mutually exclusive with symptoms such as psychosis and are a consideration in the wider context of digital health care [34].

Having a mobile phone app that was easy and quick to use was reported as a benefit of ESM and helped to integrate it into everyday life. However, a number of factors were reported to impact on the ability to integrate ESM into their day-to-day lives, including the frequency of questionnaires and notifications over the course of the day. This is in line with research reporting that completion rates can decrease when there are more items to answer [6,32,35,36]. The number of questionnaires and of questions within them is worth reflecting on for those implementing ESM.

Increasing awareness and understanding of mental health symptoms was one of the main benefits of ESM reported by participants in this study. This included learning links between mood and voice-hearing experiences, as well as triggers of such experiences. Research has found that increasing insight into mental health difficulties can be important for increased feelings of empowerment, help-seeking behaviors, and recovery [37,38]. This suggests that even as an assessment tool, ESM could be used as a way for people with psychosis to learn and understand more about their mental health experiences, which can have positive impacts on individual mental health outcomes and overall recovery.

Participants made a number of suggestions to improve their experience, including increasing customization, being able to see past responses, and access to strategies and solutions within an app. These customizations may compromise assessment with ESM, as they could influence participants’ responses; however, such suggestions are important to consider in the development of digital interventions. For example, the availability of previous entries may be beneficial in order to aid with recall in populations of people with psychosis where cognitive difficulties such as memory can be affected [39]. Including coping strategies or solutions could also be important for digital tools and interventions in supporting people to increase skills in self-management. Increased ability to self-manage has positive implications for people with psychosis in terms of increasing feelings of control, empowerment, and hope for recovery [40,41]. The incorporation of messages to enhance self-management has recently been reported within an RCT exploring the EMPOWER app, which showed promising results [42].

### Strengths and Limitations

It is important to consider the findings of this study in light of various strengths and limitations. A major strength of this study was the involvement of PPI consultants at many stages of the research in line with INVOLVE guidelines [43]. PPI involvement in research has been found to be beneficial in enhancing the quality and applicability of research, ensuring that it is user-focused, as well as helping to reduce stigma and increasing opportunities to connect with others. Despite this, PPI involvement in research is still inconsistent [44,45]. Although this is a strength of the study, it is worth noting that, for a variety of operational reasons, PPI consultants did not coconduct all 17 of the interviews, with a potential risk of bias, given some inconsistency in the interview process. Furthermore, although PPI consultants were involved at a number of stages of the research, they were not actively involved in the early stages of data analysis. Other research has reported on how PPI researchers may interpret data differently compared with academic researchers and therefore actively involving PPI consultants in the analysis of data could help to reduce bias in interpreting [46]. It is therefore important for PPI consultants to be involved in this stage in order to create meaning from data from multiple perspectives. PPI consultants were also not directly involved with the recruitment of participants. A previous review has found that involving PPI consultants with the recruitment process can increase enrollment numbers of participants [47], which may have been important in this study considering that only 17 out of the 59 participants approached were recruited for interview. It is worth noting the potential limitation related to the current sample used within the study. All of the participants had experiences of auditory hallucinations, and symptom severity was assessed based on outcomes of the PSYRATS-AH; therefore, the transferability of the findings to people who may experience other symptoms related to psychosis could be limited and would benefit from further research. Finally, this study did not examine ESM design considerations; however, previous reviews have found these to be important in enhancing ESM completion. One design consideration, in particular, mentioned in this study relates to the availability of support during ESM use, with researcher-initiated contact having been previously found to increase completion rates [48]. Further design considerations could include the number of times that the questionnaires are administered throughout the day, which could differ across studies.

### Conclusions

This study has provided important insights into the experiences of using ESM and factors that could affect engagement with this method among people with psychosis. It has also highlighted areas to consider when developing and implementing ESM and digital interventions with this population. Furthermore, the range of benefits for multiple stakeholders of actively involving PPI consultants in research has been discussed and the importance highlighted of PPI consultants’ involvement at all stages within the research process.

## Supplementary material

10.2196/78204 Multimedia Appendix 1 Experience sampling methodology debrief questionnaire.

10.2196/78204 Multimedia Appendix 2 Responses to experience sampling methodology debrief questionnaire.

10.2196/78204 Multimedia Appendix 3 Study themes and participant quotes.

## Abbreviations

EMI: ecological momentary intervention
ESM: experience sampling methodology
NIHR: National Institute for Health and Care Research
PPI: patient and public involvement
PSYRATS: Psychotic Symptom Rating Scales
PSYRATS-AH: Psychotic Symptom Rating Scales—Auditory Hallucinations Subscale
RCT: randomized controlled trial
