# Supplementary material for: Exploration of Factors That Affect Engagement With the Experience Sampling Method and Service Users’ Experience of This Within the AVATAR2 Trial: Mixed Methods Study
Source: JMIR Form Res. 2025 Dec 12;9:e78204. doi: 10.2196/78204 (PMC12700335; doi:10.2196/78204)
Supplement: Multimedia Appendix 1 [file formative-v9-e78204-s001.docx]

Multimedia Appendix 1. Experience sampling debrief questionnaire.

1. When answering the questions about the “main voice” during the experience sampling week did you answer about the same voice most of the time?

YES / NO

2. The experience sampling device disrupted my everyday life.

Not at All Very Much So

1 2 3 4 5 6 7

Any Comments?

3. The questionnaire was easy to complete.

Not at All Very Much So

1 2 3 4 5 6 7

Any Comments?

4. At times I had to rush to complete the questionnaire.

Not at All Very Much So

1 2 3 4 5 6 7

Any Comments?

5. The experience sampling device stopped me from doing my usual activities.

Not at All Very Much So

1 2 3 4 5 6 7

Any Comments?

6. I found it embarrassing when the alarm sounded around other people.

Not at All Very Much So

1 2 3 4 5 6 7

Any Comments?

7. I found it easy to remember to carry the experience sampling device with me.

Not at All Very Much So

1 2 3 4 5 6 7

Any Comments?

8. The training I received was adequate to use the device for the whole week.

Not at All Very Much So

1 2 3 4 5 6 7

Any Comments?

9. I felt supported by the research team during the experience sampling week.

Not at All Very Much So

1 2 3 4 5 6 7

Any Comments?

10. I enjoyed the experience sampling week.

Not at All Very Much So

1 2 3 4 5 6 7

Any Comments?

11. The experience sampling device changed my usual routine.

Not at All Very Much So

1 2 3 4 5 6 7

Any Comments?

12. Any general comments about the experience that you feel would benefit participants taking part in similar research in the future?
