# Supplementary material for: Exploration of Factors That Affect Engagement With the Experience Sampling Method and Service Users’ Experience of This Within the AVATAR2 Trial: Mixed Methods Study
Source: JMIR Form Res. 2025 Dec 12;9:e78204. doi: 10.2196/78204 (PMC12700335; doi:10.2196/78204)
Supplement: Multimedia Appendix 2 [file formative-v9-e78204-s002.docx]

Multimedia Appendix 2. Responses to ESM debrief questionnaire

|  | Q1.  Main voice | Q2.  Disruption | Q3.  Easy to complete | Q4.  Rush to complete | Q5.  Interrupted activities | Q6.  Embarrassed around others | Q7.  Easy to carry | Q8.  Training | Q9.  Support from team | Q10. Enjoyment | Q11. Changing routine |
| --- | --- | --- | --- | --- | --- | --- | --- | --- | --- | --- | --- |
| Yes N(%) | 152 (74.5) |  |  |  |  |  |  |  |  |  |  |
| No | 29 (14.2) |  |  |  |  |  |  |  |  |  |  |
| 1 |  | 66 (32.3) | 2 (1) | 67 (32.8) | 107 (52.5) | 139 (68.1) | 9 (4.4) | 2 (1) | 2 (1) | 5 (2.5) | 92 (45.1) |
| 2 |  | 32 (15.7) | 2 (1) | 23 (11.3) | 32 (15.7) | 12 (5.9) | 10 (4.9) | 0 (0) | 0 (0) | 9 (4.4) | 31 (15.2) |
| 3 |  | 30 (14.7) | 11 (5.4) | 14 (6.9) | 19 (9.3) | 12 (5.9) | 9 (4.4) | 5 (2.5) | 2 (1) | 13 (6.4) | 12 (5.9) |
| 4 |  | 16 (7.8) | 15 (7.4) | 26 (12.8) | 8 (3.9) | 7 (3.4) | 17 (8.3) | 5 (2.5) | 6 (2.9) | 37 (18.1) | 15 (7.4) |
| 5 |  | 14 (6.9) | 21 (10.3) | 30 (14.7) | 7 (3.4) | 4 (2) | 11 (5.4) | 12 (5.9) | 17 (8.3) | 43 (21.1) | 13 (6.4) |
| 6 |  | 14 (6.9) | 35 (17.2) | 12 (5.9) | 3 (1.5) | 4 (2) | 24 (11.8) | 19 (9.3) | 25 (12.3) | 32 (15.7) | 10 (4.9) |
| 7 |  | 9 (4.4) | 95 (46.6) | 7 (3.4) | 4 (2) | 2 (1) | 100 (49.0) | 138 (67.7) | 128 (62.6) | 41 (20.1) | 7 (3.4) |
| **Total** | 181 | 181 | 181 | 179 | 180 | 180 | 180 | 181 | 180 | 180 | 180 |

N.B. Responses to question 2 – 11 were given on a 7-point likert scale which ranged from 1 = ‘’not at all’’ to 7 = ‘’very much so’’.

Footnote:

Q1. Relates to whether the voice the participant was experiencing in that moment was the main voice they experience

Q4. Related to whether there were times that they had to rush the questionnaire when completing it

Q6. Relates to whether participants felt embarrassed answering the questionnaire when in the company of others

Q8. Relates to whether the training received prior to using ESM was sufficient

Q9. Relates to how support they felt by the research team during ESM completion

Q10. Relates to the amount they felt their routine changed as a result of ESM.

Multimedia Appendix 2. Study themes and participant quotes

| **Themes** | **Sub-themes** | **Participant quotes** |
| --- | --- | --- |
| ***Factors affecting engagement*** | Integrating ESM into daily life  Impact of symptoms  Experience with technology  Experience of therapy and/or research | *‘‘’I think it was because it was quite simple...There wasn't a lot where umm you become overwhelmed’’* (P009, line 332, Medium).    *‘’If I was travelling or walking somewhere, uh, it's not. I don't really want to be looking at my phone while I am doing M path while walking. From one place to another or on the bus or in a taxi, or if I'm shopping’’* (P016, lines 126-127, Low).  *‘’I think it's important to stress that it's only a minute, you know, although it might be pinging all the time, it's it's only a minute’’* (P008, lines 261-262, Medium).  *‘’Lots of yes and no questions, but the great thing about yes and no questions is that they can be done quickly. So I could I could tap through it all’’* (P016, lines 105-106, Low).  *‘’So when I was receiving the certain questions every day, every minute or every hour. I've just didn’t like doing it so. Well, it was the same question, so I felt a bit like bored’’* (P015, lines 80-82, High).  *‘’Yeah, I think about six days was. Was enough’’* (P008, line 194, Medium).  *‘’That's why I did it so much because I was. I thought it's only a week. Just go for it. But if it was a longer time period. I wouldn't be able to have that I wouldn't be able to sustain that amount of energy...Or interest in it’’* (P003, lines 172-173, High).  *‘’If I get, maybe if I get a visit from family...friends round or something maybe? That would. I yeah, that, that, that might make it a bit slightly more difficult for me to to keep up to answer the questions, when the when the beep goes’’* (P017, lines 150- 152, Low).  *‘’but if I was in the car, I would just wait until I got home. I mean, I did answer it eventually, just maybe not at the exact time it pinged’’* (P008, lines 208-209, Medium).  *‘’I feel like it's like A. Bit of a weight to carry when I'm out and about it's it's it's not. It's like a? whats that word when something is. Not a liability....A burden that's it’’* (P003, lines 266-268, High).  *‘’Yeah, you know, just turn the phone off. Yeah, you know what I mean? And just like, say oh the battery run out’’* (P005, lines 186-187, Medium).  *‘’I sometimes didn’t hear it ring but I was always checking in with it once an hour’’* (P014, line 53, Low).    *‘’I can't really fill this in right now because I'm not hearing any voices’’* (P007, line 180, High).  *‘’But I was a bit like set back because I thought I was just in the middle all the time and not moving on’’* (P013, lines 268-269, Low).  *‘’Yeah that really did get me for a few hours that you know what I mean? And then I just didn't think that way again, you know’’* (P005, line 225, Low).  *‘’I rarely hear voices when I'm doing the M path’’* (P016, line 185, Low).  *‘’Yeah, because you know what they're slippery ********. These voices demons. Whatever you wanna call him. And they'll know they're about to log on and do this stuff and they'll just retract themselves...And then come back as soon as you've done the app’’* (P003, lines 235-237, High).  *‘’Yeah, I can, but obviously, when I'm in that state. It's hard to remember what I've done what I've said what's being said. And so I might well have done it, but* ***(Ok)*** *because it makes me get quite chaotic can't remember if I have done it’’* (P003, lines 184-185, High).  *‘’But at times I don't remember. Where I would complete the the beep and then I would look at it and the other sections beforehand was done, but I don't I don't remember doing that’’* (P009, lines 354-356, Medium).  *‘’I think it was just about remembering to answer the questions within a certain time period’’* (P017, lines 259-260, Low).  *‘’And if I don't understand on apple. So I talk to my <relative> and <relative> is a bit more savvy than I am’’* (P006, lines 214-215, No ESM).  *‘’It's just like basics. Because I'm not very. Computer literate or anything like that’’* (P013, line 29, Low).  *‘’I mean, I'm not very sort of tech savvy at all. I don't really know how to rearrange all my files and things like that. You know what I mean?..So I just used the phone. Really. Basically’’* (P006, lines 119-122, No ESM).  *‘’Nothing, just texting and phoning’’* (P011, line 48, No ESM).  *‘’I needed a hand setting it up’’* (P011, line 77, No ESM).  *‘’Because I was thinking well I've got a phone. Yeah, you know, I mean, why they giving me a phone? You know what I mean? What they want me to answer all these things. You know what I mean? But afterwards I did. I understood straight away’’* (P005, lines 256-258, Medium).  *‘’this technologies makes you very hard you for your life,* ***(right OK)*** *that's why. That's why they don't want anything there. I don't have TV. I don't want nothing. I don't. I don't want technology at all.* ***(Yeah)*** *you see you just keep yourself away from them. That's why I don't want to use them’’* (P010, lines 221-223, No ESM).    *‘’It was good to be helping as well as being helped’*’ (P014, line 72, Low).  *‘’I wouldn't say no, I'll give it a try’’* (P003, line 306, High).  *‘’I'm looking for some things to help me. I don't want anything. I just want help’’* (P005, line 71, Medium).  *‘’Yeah, I'll just talk whatever I have to do in order to...help me to get through this difficult time with my condition of psychosis’’* (P017, lines 102-103, Low).    *‘’Well, I could see what <therapist> was trying to achieve...So I kind of understood the necessity to answer it’’* (P008, line 218, Medium). |
| ***Perceived benefits of ESM*** | Access to increased support  Enhanced awareness | *‘’It calmed the voices down’’* (P014, line 163, Low).  *‘’It made it more simple. Yes it made me more less stress with the voices’’* (P014, line 145, Low).  *‘’So in in a way it was kind of trying to find out how what the voices were like and how how it made you feel and...what you could do is pinpoint exactly what you should do more of and what you should do less of’’* (P008, lines 107-109, Medium).    *‘’But that that that idea was there, but I just kind of, but I've had that for a long time and it hasn't manifested into reality so that's what I tell myself that it wasn't real last time probably not gonna be real this time’’* (P003, lines 34-36, High).    *‘’I do think that it did help at times...to be more aware because I wasn't really aware of how frequently, frequently like I would hear <voice> … because I don't think about that so being able to think’’* (P009, lines 345-347, Medium).  *‘’it did help answering them because like for me it made me think’’* (P005, lines 276- 277, Medium).  *‘’it would kind of bring me back to the present in a way because like, if I was off in my world and then the app would beep, then it's time to do my things’’* (P007, lines 98- 99, High).    *‘’I remember mm whenever the beep went off. It was more so I could take time to.* ***(Umm)*** *sit down and think about. How the voices impacted and had an impact not on in the day like right now in the moment’’* (P009, lines 57-58, Medium).  *‘’For you to realise yourself...what you're doing, what you're thinking, how busy you are. You know what? When? And this is quite effective when you actually do it yourself, you begin to realize that what you're doing to avoid what you're doing when the voices are worse’’* (P008, lines 51-53, Medium).  *‘’there may be like a correlation between what you're doing and the voices’’* (P001, line 320, High).  *‘’It also, the app made me see that I'm doing quite well because. I was I wasn't being over overpowered by these voices. You know, although they really, really annoying and **** me off. But I I wasn't in. Like I realised how many times, I selected that they don't have control over me’’* (P003, lines 197-199, High). |
| ***Suggestions for improvement*** | Specificity and customisation  Need for further support  Access to solutions and strategies | *‘’I think it it could ask if you are hearing voices and then just say. Give a brief summary of what they are.* ***(Uh-huh)*** *And where you are...offer automatic grounding techniques like where are you take notice of the surroundings...where are you who are you with? And what can you do right now to help yourself?’’* (P003, lines 189-191, High).  *‘’..like when it says on the last one about hearing voices, are you hearing voices at the moment. I wasn’t sure whether it should be in that exact minute that I look at the app or should it be like for the last hour’’* (P003, lines 215-217, High).  *‘’Maybe say you are driving between...I don’t know 3 and 4. Or I don’t know if you kind of say I’m not gonna be able to answer it at this time’’* (P008, lines 467-468, Medium).  *‘’What what I want from it is, I want to know that someones listening to it. It's actually being used. Like ideally it'd be used for my doctor or by my research or my avatar therapy partner’*’ (P016, lines 63-65, Low).    *‘’I mean, you know, maybe I was a bit unsure about how I should fill in the the, the, the, the, the questions given that it wasn't related to my experience. I I know that there's meant to be a bit of a blind spot with the experiment so that I didn't communicate what I was doing to the researcher at the end’’* (P001, lines 86-88, High).  *‘’Yes, I think. That I think the thing that would make it even more valuable is if someone was monitoring negativity for safety....some of the questions could? Between between one session on the app to another session on the app you might have a dip in your mood.* ***(Uh-huh)****. Uh, and it some people who hear voices are suicidal. Uh, so it would be good monitor this for safety monitor people that have a dip in their..reporting self-reporting. To safety check them to do a a welfare check if if there. Umm. If the mood is taking a dip or drop because of the voices’*’ (P016, lines 51-55, Low).  *‘’So it was quite easy to just check the boxes on my phone than on a piece of paper but with the piece of paper you you're able to see how your moods are* ***(Umm)*** *the voices have impacted you are but in app you weren't able to...umm see your past logs’’* (P009, lines 318-320, Medium).  *‘’I would remember it longer. More helpful with memories of it’’* (P014, line 214, Low). |
